# In Vitro Evaluation of Dimensional Stability of Alginate Impressions after Disinfection by Spray and Immersion Methods

**DOI:** 10.5681/joddd.2010.032

**Published:** 2010-12-21

**Authors:** Fahimeh Hamedi Rad, Tahereh Ghaffari, Sayed Hamed Safavi

**Affiliations:** ^1^ Assistant Professor, Department of Prosthodontics, Faculty of Dentistry, Tabriz University of Medical Sciences, Tabriz, Iran; ^2^ Dentist, Private Practice, Tabriz, Iran

**Keywords:** Dimensional stability, alginate, disinfection

## Abstract

**Background and aims:**

The most common method for alginate impression disinfection is spraying it with disinfecting agents, but some studies have shown that these impressions can be immersed, too. The aim of this study was to evaluate the dimensional stability of alginate impressions following disinfecting by spray and immersion methods.

**Materials and methods:**

Four common disinfecting agents (Sodium Hypochlorite, Micro 10, Glutaraldehyde and De-conex) were selected and the impressions (n=108) were divided into four groups (n=24) and eight subgroups (n=12) for disinfecting by any of the four above-mentioned agents by spray or immersion methods. The control group (n=12) was not disinfected. Then the impressions were poured by type III Dental Stone Plaster in a standard method. The results were ana-lyzed by descriptive methods (mean and standard deviation), t-test, two-way analysis of variance (ANOVA) and Duncan test, using SPSS 14.0 software for windows.

**Results:**

The mean changes of length and height were significant between the various groups and disinfecting methods. Regarding the length, the greatest and the least amounts were related to Deconex and Micro 10 in the immersion method, respectively. Regarding height, the greatest and the least amounts were related to Glutaraldehyde and Deconex in the im-mersion method, respectively.

**Conclusion:**

Disinfecting alginate impressions by Sodium Hypochlorite, Deconex and Glutaraldehyde by immersion method is not recommended and it is better to disinfect alginate impressions by spraying of Micro 10, Sodium Hypochlorite, Glutaraldehyde and immersion in Micro 10.

## Introduction

Undoubtedly, alginate is one of the most popular materials for making an impression of the mouth. Considering the prevalence of some diseases like hepatitis B, immune deficiency syndrome and new wave of drug resistant tuberculosis, the clinicians are required to exercise caution and disinfect this material. Dental impression is certainly one of the ways for transmission of pathogens from the office to other environments.^1^ Therefore, all the alginate impressions should be disinfected before being poured with gypsum. The most common method for disinfection is spraying the disinfecting agents on alginate impressions, but some studies have shown that these impressions can be disinfected by immersion method as well.^2,4,5,6,7^

Wu et al^3^ carried out a study on the disinfection of impression materials and stone casts with a new method called “ultrasonically nebulized electrolyzed oxidizing water” in dental clinics. Their results revealed no significant differences in dimensional changes between the control group and the group tested. But the dimensional accuracy of both groups was more than sodium hypochlorite-disinfected group, which was statistically significant.

The aim of this study was to evaluate the dimensional stability of alginate impressions following disinfecting by spray and immersion methods, so that the proper method of disinfecting the impressions concerning disinfecting agent and its method could be determined.

## Materials and Methods

In this in vitro study, 108 alginate impressions were taken from a metallic laboratory model using metallic perforated trays.

The model used was made of bronze by an accurate milling machine with an accuracy of 0.02 mm; it measured 50х50х20 mm and consisted of two parts:

- A bottom or the lower part with two holes for securing the model to the table. This part protrudes 1 mm from the body sides of model to be distinguished from the body. The thickness of this portion is 5 mm (the overall bottom dimensions are 52х52х5 mm).
- Body portion of the model: This cubic part is on the bottom portion with dimensions of 50х50х20 mm (Figure 1a).

Twenty-four perforated trays with a thickness of 1 mm and a dimension of 60х60х30 mm were made using galvanized iron. For equality of the distance of tray from the walls of the model, one stop with a height of 5 mm was designed in the middle of the tray. A handle was made on the upper side of the trays for easy control and manipulation the trays (Figure 1b).


Figure 1. Metallic laboratory model (a), and metallic perforated tray (b).

**a:**
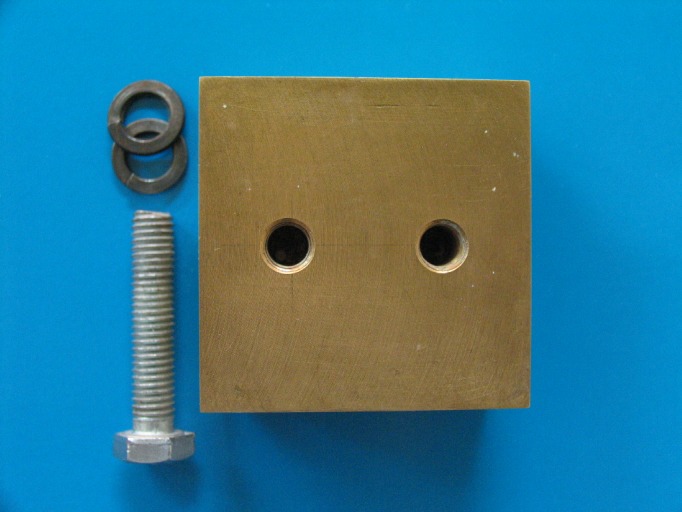

**b:**
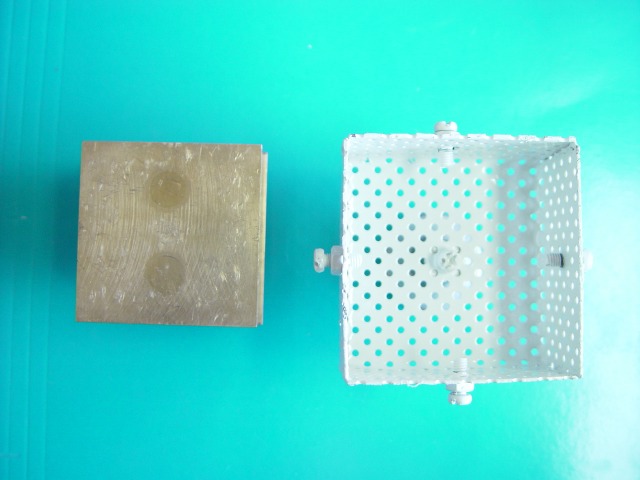

Normal-setting alginate (Zermak Co., Italy) was used as the impression material. First, alginate adhesive (Dentires S.A Co.) was sprayed inside the trays and put away for 15 minutes until it dried up. The alginate was mixed for 30 seconds based on manufacturer’s directions using a special spoon for water and powder (3 spoonfuls of powder and 3 spoonfuls of water for each tray), and then some part of it was placed on the model and the rest in the tray; finally the impressions were made.

One hundred and eight alginate impressions were randomly divided into 8 test groups (n=12) for disinfecting by any of the four disinfectants by spray or immersion methods. The control group (n=12) was not disinfected.

All of the impressions were placed under cold running water for 30 seconds immediately after removal from the model, and then the excess water was removed with air spray in a way that the surface would not be desiccated. Then the impressions were disinfected for 8 minutes and again placed under running water for 30 seconds. The control group was first rinsed under cold water for 30 seconds and then protected in a wet environment for recovery lasting 8 minutes^2^ and finally rinsed under running water for 30 seconds again. Disinfecting agents included:

- 5.25% Sodium Hypochlorite (Paksan Co., Iran), available on the market and used in this study without diluting
- 2% Glutaraldehyde (Behsa Co., Iran), used without diluting
- Deconex (Irenic Company Switzerland), used without diluting
- Micro 10, diluted to 1/10 concentration according to manufacturer’s recommendations

The plaster used was Moldano (Pars Dental Co., Iran). It was measured and mixed according to manufacturer’s recommendations (100 g powder + 18 g water).

After mixing for 30 seconds, the plaster was poured inside the impression gently by vibrating; it was dislodged from the impression after 45 minutes. Under the plaster specimens its number and group were written. The length and height of the laboratory models and the plaster specimens were measured after 24 hours.

Since the length and width of the original model were equal (50 mm), in specimen measurement the average of length and width were considered only as length. Measurements were carried out by two observers.

A digital caliper instrument (L.G. Co., China) with a precision of 0.02 mm was used for specimen measurements.

Kolmogorov-Smirnov test showed normal distribution of data. Data were analyzed by descriptive statistical methods (mean and standard deviation), t-test and one- and two-way analysis of variance (ANOVA) using SPSS 14.0 software for windows. In this study statistical significance was set at p<0.05. Maier error test was used for personal error evaluation between the two measurements.

## Results

The dimensions of casts measured by the digital caliper are reported in Table 1. Means ± standard deviations were calculated for each disinfecting agent in both experimental and control groups.

**Table 1:** Comparison of length and height between different disinfecting agents and methods

Variable	Method	Mean ± Standard deviation	t value	Degree of freedom	P-value
Sodium Hypochlorite					
Length	Spray	50.235 ± 0.07	?5.40	22	0.000
	Immersion	50.331± 0.05			
Height	Spray	20.108 ± 0.12	0.06	22	0.950
	Immersion	20.105 ± 0.20			
Glutaraldehyde					
Length	Spray	50.260 ± 0.06	?1.45	22	0.153
	Immersion	50.284 ± 0.06			
Height	Spray	20.061 ±0.10	?4.85	22	0.000
	Immersion	20.198 ± 0.09			
Deconex					
Length	Spray	50.270 ±0.06	?12.37	22	0.000
	Immersion	50.571 ±0.10			
Height	Spray	19.941 ±0.23	?1.26	22	0.215
	Immersion	20.027 ±0.24			
Micro 10					
Length	Spray	50.259 ±0.05	0.19	22	0.850
	Immersion	50.251 ±0.20			
Height	Spray	20.125 ±0.07	?0.04	22	0.969
	Immersion	20.126 ±0.08			

Analysis of the results by two-way ANOVA showed that differences in the mean length and height variations were statistically significant between disinfecting agents and methods (p<0.0005). The maximum mean of length changes was related to Deconex followed by Sodium Hypochlorite, Glutaraldehyde and Micro 10 in immersion method (Figure 2). Two-by-two comparisons of mean differences with Duncan test showed significant differences in the means of Deconex and Sodium Hypochlorite with other groups (p<0.05). However, the mean variations of length in all the groups with the controls were not statistically significant in spraying.


Figure 2. Comparison of mean length (a) and mean height (b) in disinfecting agents with disinfection method and control group.

**a:**
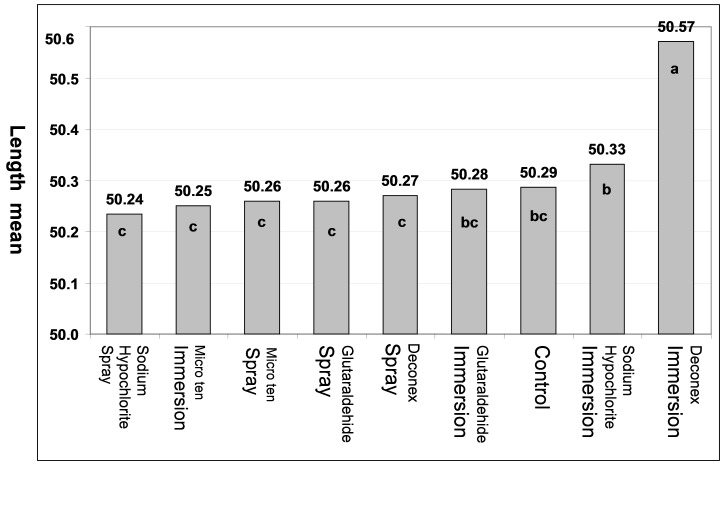

**b:**
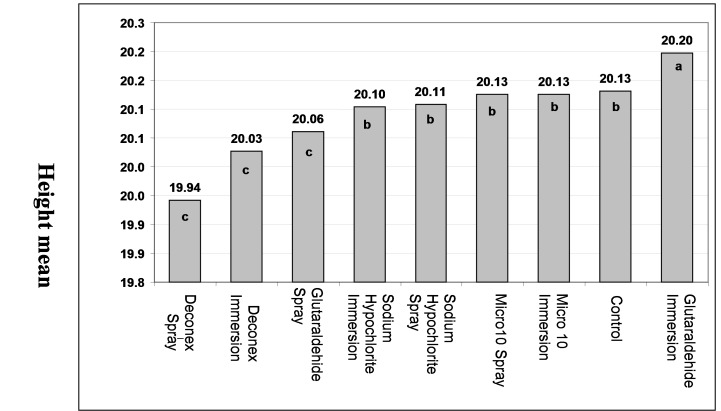

The maximum mean of height changes was related to Glutaraldehyde followed by Sodium Hypochlorite, and Micro 10 in immersion method (Figure 3). Deconex with the least mean height had statistically significant differences with other groups (p<0.05). In spraying, the mean height in Deconex with the least amount had statistically significant differences with other groups (p<0.05) (Figure 2). The mean personal error was 0.002 mm between the two observers.


Figure 3. Comparison of disinfecting agents and the control group in spraying (a) and immersion (b) methods.

**a:**
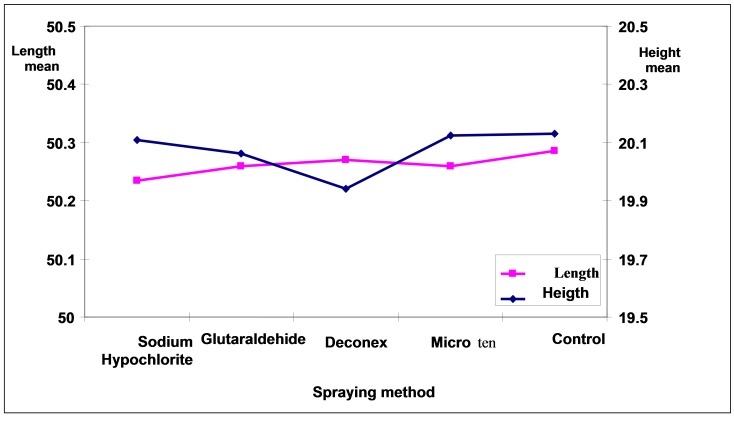

**b:**
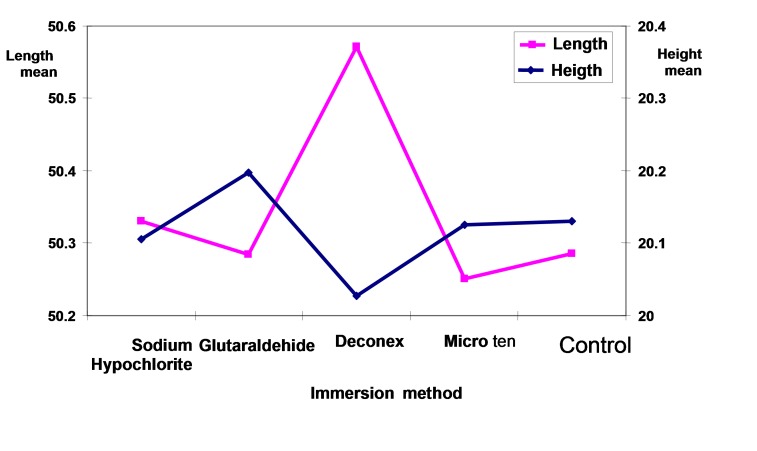

## Discussion

The conclusions drawn from studies on dimensional stability of the impressions disinfected cannot be analytically evaluated because the laboratory studies are different considering specimen dimensions, baseline measurements, and method of measurement and reporting. A common method accepted by researchers must be established and the developed technology might be useful if more precise evaluations and direct data comparisons are required.

Dimensional changes produced by chemical disinfection are not likely to affect the clinical performance. Therefore, chemical disinfection is almost harmless. However, it is supposed that some restrictions concerning duration and the method of disinfection must be applied, so that the dimensions and surface of the impression are preserved to provide effective microbial elimination. The restrictions mentioned concern the chemical nature of the materials.

The influence of disinfection on the dimensions of impressions might seem limited, but it was often obvious and in many cases was found to originate from imbibition of water. Such an effect is easily seen in the behavior of the materials, which are vulnerable to a wet environment, i.e. hydrocolloids and polyether. The imbibitions characterizing the hydrocolloids have resulted in restrictions with regard to time of immersion. It should be noted that the majority of researchers apply disinfection times of no more than 30 minutes.^8-11^ According to Taylor et al,^11^ a 10-min imbibition can be beneficial, because it works against the synergies-associated shrinkage. Instead of immersion, spraying has been suggested in many cases, but not in all^8^of the reports since it limits the exposure in the wet environment and produces remarkably stable impressions^9,12^and precise casts.^13,14^ However, spraying that is performed with reduced contact time may restrict the effectiveness of disinfection, particularly for the porous hydrophilic hydrocolloids, where microorganisms can penetrate through the body and survive in the impression.^15^ It was determined that the incorporation of disinfectants into the hydrocolloid powder^2,16^or mixing water^17^provides an effective means of additional decontamination, without leading to adverse effects considering stability^18^and accuracy.^19^

In the present study, variations of length in disinfected specimens with 5.25% sodium hypochlorite in the two methods were statistically significant, but the height variations were not significant. The results of our study were similar to those of Wu et al^3^ and Oderinu et al.^4^ Of course, it should be pointed out that the concentration of sodium hypochlorite in their study was 1%, but in ours it was 5.25%.

Rueggeberg et al^13^ disinfected alginate impressions with spraying of sodium hypochlorite and reported distinct dimensional variations,which are consistent with our results.

In the research carried out by Taylor et al^11^on dimensional accuracy of disinfected alginate impressions by sodium hypochlorite, the dimensional variation in impressions was not significant, which is not consistent with our study because of the difference in methods regarding the time of preservation of impressions and sodium hypochlorite dilution.

In our study, variations of height in disinfected specimens with 2% Glutaraldehyde in two methods were statistically significant, but the length variations were not significant. Lu et al^6^and Jones et al^7^ have reported similar reports, too.

According to our study, the mean maximum length was related to Deconex in immersion method. Since no similar studies have been carried out, it seems that the use of Deconex by immersion method is not acceptable because it causes dimensional changes in alginate impressions.

Regarding Micro 10, variations of height and length in disinfected specimens with Micro 10 in the two methods were not statistically significant. There is no published study on the effect of Micro 10 on dimensional stability of alginate impressions.

The differences in the results yielded by Micro 10 and other disinfectants in our study can be attributed to the lower disinfection efficiency of Micro 10 in comparison with other disinfectants. Therefore, at equal disinfection time, Micro 10 has the least efficacy to penetrate into the impression and produces dimensional changes in it.

In the present study, specimens disinfected by spray method showed no significant variations in length, which is consistent with the results of studies by Oderinu et al,^4^ Juggar et al,^20^ Lu et al,^6^ and Habu et al.^21^

## Conclusion

Based on the results of the present study, the use of immersion disinfection method by 5.25% Sodium hypochlorite and Deconex and 2% Glutaraldehyde is not advised because they cause dimensional changes in impressions.

Finally, within the limitations of this study, it is recommended to disinfect alginate impressions by spraying of Micro 10, Sodium Hypochlorite, Glutaraldehyde and immersion in Micro 10.
